# Mirrorless MEMS imaging: a nonlinear vibrational approach utilizing aerosol-jetted PZT-actuated fiber MEMS scanner for microscale illumination

**DOI:** 10.1038/s41378-023-00646-5

**Published:** 2024-01-22

**Authors:** Wei-Chih Wang, Ming-Yao Li, Kuan-Chang Peng, Yi-Feng Hsu, Benjamin Estroff, Pao-Yun Yen, David Schipf, Wen-Jong Wu

**Affiliations:** 1 Department of Power Mechanical Engineering, National Tsing Hua University, No. 101, Section 2, Kuang-Fu Road, Hsinchu, Taiwan 30013 China; 2 Institute of NanoEngineering and Microsystems, National Tsing Hua University, No. 101, Section 2, Kuang-Fu Road, Hsinchu, Taiwan 30013 China; 3 Department of Mechanical Engineering, University of Washington, Seattle, WA 98195 USA; 4 Department of Electrical Engineering, University of Washington, Seattle, WA 98195 USA; 5 Department of Engineering Science and Ocean Engineering, National Taiwan University, Taiwan, China

**Keywords:** Micro-optics, Electrical and electronic engineering, Micro-optics, Micro-optics

## Abstract

This study introduces a novel image capture and lighting techniques using a cutting-edge hybrid MEMS scanner system designed for compact microscopic imaging. The scanner comprises a tapered optical fiber waveguide and innovative aerosol-jet printed PZT (lead zirconate titanate) bimorph push-pull actuators on a stainless-steel substrate, effectively addressing issues that are commonly associated with PZT on silicon substrates such as fracture and layer separation. By leveraging nonlinear vibration, the scanner achieves a spiral scan pattern from a single signal input, in addition to the expected two-dimensional scanning and target illumination from two phase-shifted inputs. This capability is further enhanced by a novel process to taper the optical fiber, which reduces illumination scattering and tunes the fiber to the resonant frequencies of the scanner. The precisely tapered tip enables large fields of view while maintaining independent 2-axis scanning through one-degree-of-freedom actuation. Experimental validation showcases the successful generation of a spiral scan pattern with a 60 μm diameter scan area and a 10 Hz frame rate, effectively reconstructing scanned images of 5 μm lines, cross patterns (15 μm in length with a 5 μm gap), and structures of a Psychodidae wing.

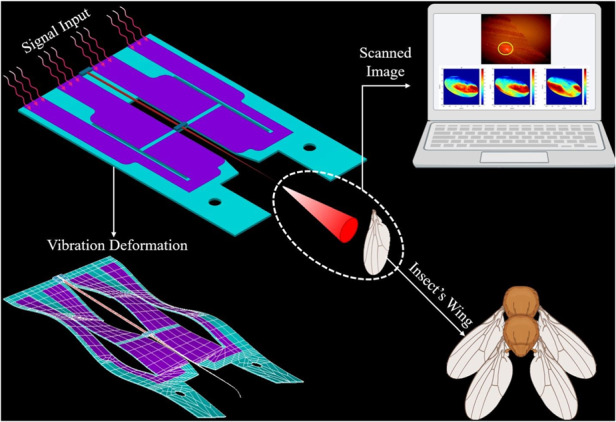

## Introduction

Miniature scanners are integral components of modern image acquisition and display technologies and have a rich history of development and use in diverse applications. Peterson introduced the first optical microelectromechanical system (MEMS) scanner in 1980, which incorporated mirrors for optical scanning^1^. Since then, miniature scanner technologies have been employed in numerous fields, including medical imaging, video projection, wearable displays, and light detection and ranging (LIDAR) for terrain mapping^2–32^.

Most commercial miniature scanner technologies currently rely on MEMS scanning mirrors, which often include complex designs and require precision components^1,5,13,15,19,32,33^. To ensure efficient actuation and deflection, the size of the scanning mirror needs to be larger than the diameter of the incident light beam to avoid issues of clipping or diffraction. This limitation on the mirror size makes reducing the overall footprint of the display system difficult, as the size is directly proportional to the resolution and/or field of view (FOV) of the device.

MEMS scanning micromirrors can be implemented using a variety of actuation methods, including electrostatic^33–35^, electromagnetic^3,36,37^, electrothermal^38–40^ and piezoelectric^41–52^ designs. Electrostatic actuators are relatively easy to fabricate and offer straightforward actuation but require higher driving voltages to compensate for their low actuation force. Electromagnetic scanners can mechanically rotate across large angles but often require bulky magnets that are difficult to integrate with compact packaging. Electrothermal actuators offer improved optical performance and have simpler fabrication methods that are compatible with integrated circuit (IC) processes, but they are also dependent on the ambient temperature and may require relatively large operating voltages or currents. Piezoelectric actuators strike a balance between compact design and low power/low voltage actuation, while offering the advantage of high dielectric strength^41–52^. However, piezoelectric actuator fabrication can be complicated, typically relying on techniques such as sol-gel, chemical vapor deposition, granule spray in vacuum, aerosol deposition or sputtering^53–66^. Additionally, current piezoelectric actuators are typically limited to small displacements in the range of 10 pm to 100 µm. Despite these issues, piezoelectric actuators are widely used in MEMS scanner technologies due to their unique combination of performance characteristics.

Our team has been conducting extensive research on MEMS optical scanning for the past two decades, including the development of a 2D scanner utilizing off-the-shelf lead zirconate titanate (PZT) actuators to control a microfabricated hybrid waveguide^20–22,25,44,45^. While the scanner exhibited predictable waveguide motion and easily controlled illumination, its fabrication process was difficult to replicate. We then developed a system that integrated the microfabricated waveguide with an electrostatic push-pull actuator and used batch processing for improved production quality and consistency^26,35^. However, the numerous steps involved in that fabrication process made production of the corresponding devices slow and costly.

**Fig. 1:**
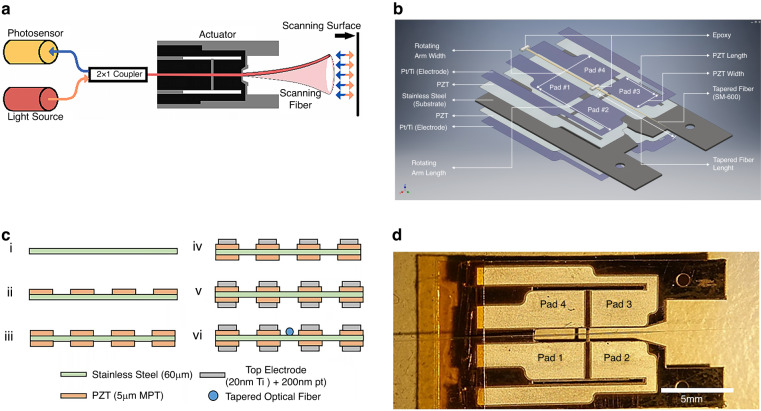
Overview of MEMS scanner concept, design, fabrication, and completed device. **a** Concept for the proposed MEMS image acquisition scanner. Orange indicates the direction of source light; blue indicates the reflected light collected by the scanning fiber. This paper only discusses the control and illumination of the scanning fiber. **b** Diagram of the “push-pull” scanner. **c** Cross section of fabrication process flow: (i) stainless steel preparation, (ii, iii) aerosol deposition of PZT (patterning by lift-off process), (iv, v) Ti/Pt electrode deposition by lift-off process, (vi) attachment of tapered optical fiber with epoxy. **d** Photograph of the completed scanner; dimensions 17 × 10 mm

To address these issues associated with current micromirror-based MEMS scanner designs and our previous scanner development efforts, our team proposes a novel 17 × 10 mm 2D scanner utilizing single degree-of-freedom (DOF) actuators driving a resonant optical fiber, as illustrated in Fig. 1a, b. This design utilizes the deflection of the optical fiber tip to create scanning motion, simplifying the overall design, reducing production costs, and increasing system flexibility. Light coupled into the scanning fiber illuminates the targeted area in a known pattern, with a photodetector mounted in parallel with the light source to receive the reflected light via a 2 × 1 coupler. Importantly, the image resolution is determined by the light spot diameter on the illumination plane, not the photodetector size. We henceforth present the development of the MEMS scanner, control of the fiber scanning pattern, and transmitted illumination image reconstruction.

## Results

### Design concept and linear operation

The design of the scanner is shown in Fig. 1b. It is a hybrid MEMS structure composed of aerosol-jetted PZT bimorph push-pull actuators patterned on a ductile 301 stainless-steel core to realize large bending displacements when excited by opposing electric fields. By using 301 stainless steel (Young’s modulus 190 GPa) instead of fused silicon <100> (Young’s modulus 130 GPa) as the substrate and central electrode, the device’s deflection and operational lifespan are improved^67,68^. When activated, the five-layer structure (cross-section shown in Fig. 1c) flexes and twists a central cross bar, inducing vibration in the securely attached tapered fiber optic waveguide and facilitating illumination of a target area for compact image capture.

**Fig. 2:**
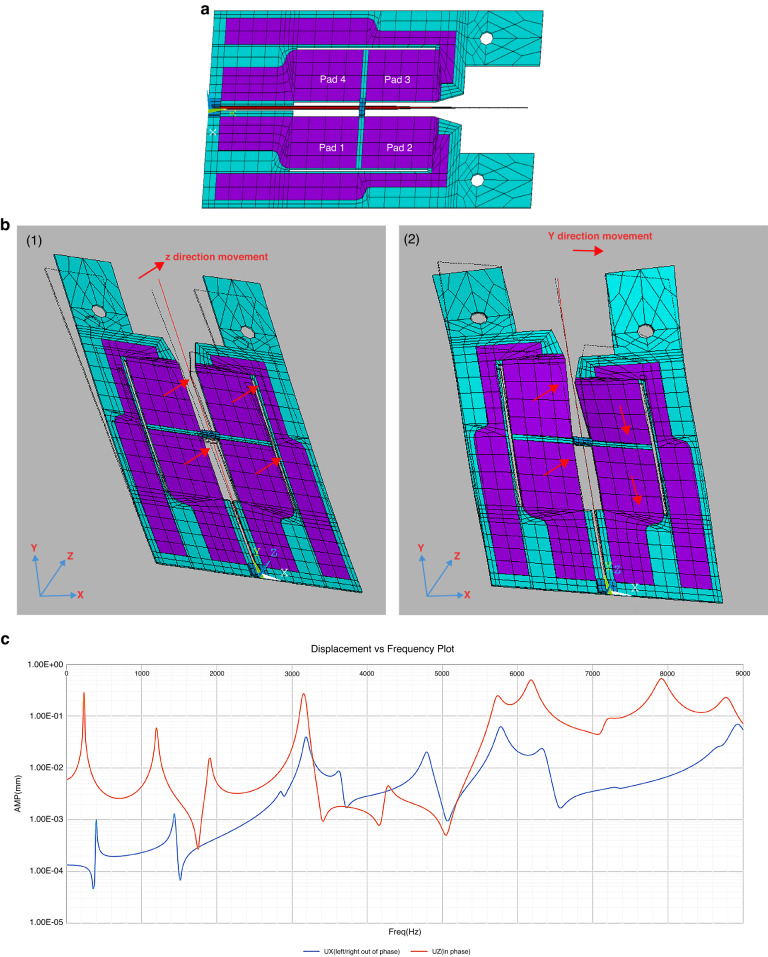
FEA model of the scanner, showing fiber actuation and simulated fiber tip displacement. **a** The FEA model (ANSYS APDL) consisting of substrate (blue), PZT thin film (purple), and tapered optical fiber (red). The boundary conditions are set to 0 displacement and rotation at the left-hand side of the device, and at the mounting holes on the right. **b** FEA mode shapes showing 2D motion excited by one-dimensional actuation: (1) Vertical fiber motion @ 3189 Hz. (2) Horizontal fiber motion @ 3190 Hz. **c** Simulated vertical and horizontal displacement from 0–9 kHz with a tapered fiber tip. (Ux: Horizontal, Uz: Vertical displacement)

The four PZT pads enable a novel push-pull actuation mechanism, translating one-degree-of-freedom (DOF) actuation into two-dimensional motion while maintaining independence of the axes in the two-axis scanning motion. The viability of this concept was successfully demonstrated by finite element analysis (FEA) of the scanner, as shown in Fig. 2a, b.

Upon activation of the pads, in- or out-of-phase oscillations occur in the vertical plane, resulting in both vertical and horizontal movements of the fiber waveguide. For vertical motion, all the pads (Figs. 1b, d and 2a) move in the same direction with equal magnitude and phase. This vertical movement transfers to the cross arm and the waveguide, as shown in Fig. 2b(1). Horizontal motion is generated by the pair of pads on each side of the fiber (i.e., #1 and #3 vs. #2 and #4), moving with the same direction, magnitude, and phase but 180° out of phase with the other pair. The opposed bending motion of the actuators induces twisting in the cross arm, resulting in horizontal movement of the waveguide, as depicted in Fig. 2b(2).

### Device modeling

FEA was performed using ANSYS Mechanical APDL to simulate the behavior of the PZT actuator and optimize the optical fiber parameters. To simplify the FEA model, the relatively thin top electrode layer (the thickness is <0.5% of the bottom electrode) is neglected. The final FEA model consisted of a 60 μm patterned stainless-steel substrate, a 5 μm PZT layer, and a tapered optical fiber epoxied to the cross arm of the device. ANSYS element types SOLID186, SOLID226, and BEAM189 were used to model the stainless-steel substrate, PZT film, and tapered optical fiber, respectively. The boundary conditions were set to zero displacement and rotation in the X, Y, and Z planes at the proximal base edge (electrode contact) side and at the distal circular holes.

The material properties used in the simulation are listed in Table S1 of the Supplemental Information. The permittivity (ε) of PZT was set to 8.854 × 10^−12^ F/m. The nonzero piezoelectric constants and nonzero elements of the compliance matrix are listed in Table S2. A harmonic analysis was performed with a +10 V amplitude triangular wave applied to the PZT actuator using a linear elastic model without residual thermal stress from fabrication. The damping ratio was fit to 0.0115 based on the experimental results, which is slightly higher than the estimated value based on previous air viscosity experiments^69,70^. The overall damping of the system is difficult to determine from simulations because it includes contributions from both structural/material damping and air damping.

The simulated resonant frequencies of the system, push-pull actuator, and tapered fiber are listed in Table 1. Frequencies up to 10 kHz were investigated, as higher frequencies result in relatively small displacements and harder-to-obtain higher order modes. Figure 2 shows the typical mode shape of the scanner, as well as the first bending mode of the tapered fiber in the horizontal and vertical directions at frequencies of 3190 Hz and 3189 Hz, respectively.

**Table 1:** Modal analysis results

Mode	Full System (Hz)	Actuator (Hz)	Static etched fiber (Hz)	Quasistatic etched fiber (Hz)	Mode	Full System (Hz)	Actuator (Hz)
1	238	224	1271.3	3188	13	5729	7162
2	402	402	1276.3	3190	14	5778	7328
3	1206	1203	7983.2	5774	15	6186	7952
4	1444	1444	8016.1	5779	16	7171	8680
5	1908	1906	10536	8907	17	7329	10109
6	2872	2872	10560	8929	18	7917	10461
7	3152	3141	22849	10500	19	8688	10564
8	3189	3647	22934	10531	20	8790	12116
9	3190	4302	29623	13170	21	8925	
10	3647	4801		13197	22	9818	
11	4268	6197			23	10332	
12	4801	6350					

The displacement of the distal end of the fiber serves as an indicator for the FOV of the device. Harmonic and transient analyses were performed to obtain the waveguide movement at specified operating frequencies. A static parametric study of the actuator portion was conducted to identify suitable design dimensions, followed by harmonic analysis of selected dimensions for verification. The width and length of the rotating arm and the PZT pad were also investigated because these factors influence the tip displacement. Pads 1 and 3 were actuated in phase by an applied voltage of 10 V, with other dimensions based on the previously optimized waveguide simulation. The maximum driving voltage for the PZT actuator was set at 20 V based on a poling voltage of 34 V, which was found to produce the best electromechanical coupling coefficient among the tested samples. Based on the optimized parameters for matching the fiber and actuator frequencies, the harmonic results for horizontal and vertical displacement of the fiber distal end are shown in Fig. 2c.

### PZT actuator fabrication

The development of the scanner involves several unique fabrication approaches and materials not commonly found in previous reports on MEMS production, the most novel of these being the use of stainless steel instead of the typical semiconductor substrate as the base material. This decision offered significantly larger deflection and enhanced mechanical support compared to the more traditional semiconductor substrates. Another distinctive approach was the use of aerosol deposition. This method, characterized by its simplicity, time-saving attributes, and efficiency successfully produced high-quality PZT films at room temperature, making the entire fabrication process both cost-effective and time-efficient^71^.

**Fig. 3:**
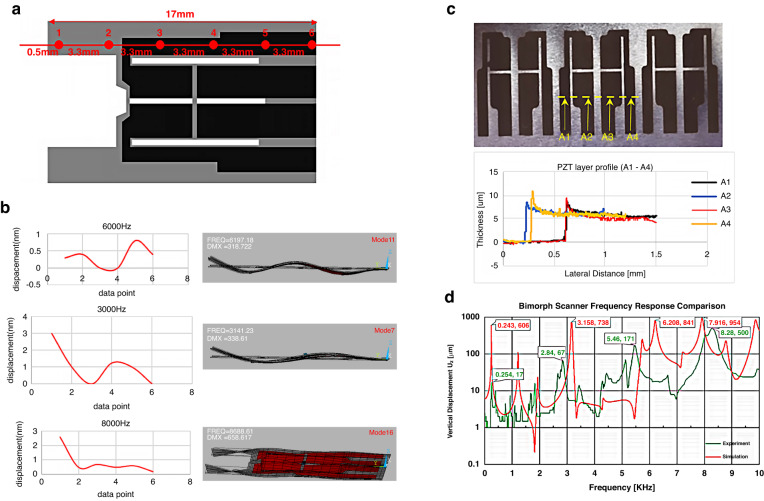
Actuator profile measurement, responsivity, and frequency response. **a** Diagram of measurement sites recorded by laser Doppler vibrometer. **b** (Left) Reconstruction of the actuator vibration obtained from laser vibrometer measurements at the points in **a**. (Right) The mode shapes generated from the ANSYS simulation, showing agreement between simulation and measurement. **c** PZT thin film thickness measured using a profilometer at sites A1–A4. **d** Comparison of vertical displacement (Uz) between simulation and experimental results

The scanner fabrication process (Fig. 1c) includes both surface and bulk micromachining techniques. The devices are fabricated on a 60 μm 301 stainless-steel substrate, which serves as both the central electrode and primary support for the scanner (Fig. 1b, c). The substrate was cleansed with piranha solution (H_2_SO_4_: H_2_O_2_ = 3: 1) to remove organics, rinsed with DI water (Fig. 1c(i)) and dried. Next, a 75 μm layer of THB-151 N negative photoresist (JSR Corporation) is spin-coated onto both sides of the substrate. These relatively thick layers of photoresist are necessary to withstand the high velocity of the MPT-Grade PZT powder (Hayashi chemical Industry Co., Ltd., HIZIRCO PZT Series) used in the AD process. This scheme ensures uniform film thickness and smooth sidewalls for the deposited PZT films (shown in Fig. 3c). The photoresist layers are patterned through a mask with 365 nm irradiance and developed in a 2.38% TMAH solution using a double-sided aligner (ABM, Inc.) to ensure precise alignment of the front- and back-side patterns.

During fabrication, PZT is deposited at approximately 1.5 μm/hour to create four 2596 × 3575 μm x ~5 μm thin film pads (Fig. 1c(ii))^24^. The procedure is repeated for the back side of the substrate before removing the remaining photoresist with acetone (Fig. 1c(iii)). Successively depositing the PZT layers results in balancing of the residual thermal stress, keeping the substrate flat for electrode deposition.

The Ti/Pt electrodes are fabricated using a Peva-450I E-beam evaporation system (AST) with the lift-off technique (Fig. 1c (iv), (v)). For the fabrication of these electrodes, SPR220-7 positive photoresist (Dow Chemical Company, MEGAPOSIT SPR220 Series Positive Tone Photoresist) is utilized. A 7 μm layer of photoresist is applied for the top electrode, and an ~10 μm layer is applied for the bottom electrode. These layers are patterned through i-line UV exposure and developed in a 2.38% TMAH solution. The use of SPR220-7 photoresist ensures sharply defined edges and corners, preventing shorting between the electrodes. After patterning, the photoresist is removed, and the electrodes are patterned using E-beam evaporation. This process is repeated on the reverse of the device to fabricate the bottom electrodes (Fig. 1c(iv,v)). The final etching process involves the use of aqua regia (50% chlorotic acid solution, H_2_O: HNO_3_: HCl = 4: 1: 3) on a stainless-steel substrate. This etching step yields the middle electrode geometry and releases the final device (Fig. 1b, d). The device is annealed with a ramp-up rate of 2.1 °C/min for 4 h to 520 °C, held for three hours before and gradually cooled to room temperature over the next 25 h (see Fig. S1). The annealed device is poled at 10 V/µm on a 180 °C hot plate. While each pad has a slightly different measured capacitance, the values for the average relative dielectric constant before (ε_r_ = 1385) and after (ε_r_ = 1276) poling the scanner are remarkably close to the manufacturer’s value of ε_r_ = 1300 (calculations provided in Supplementary Information). Finally, polyvinyl acetate glue is applied to secure the fiber to the central actuator arm (Fig. 1b, c(vi), d).

### Actuator frequency response tests

Frequency response tests were conducted to determine the natural frequencies of the actuator and establish the operational frequency limits of the device prior to the installation of the optical fiber. The tests apply a ±10 V triangle wave signal to all eight pads of the actuator, sweeping from 1 kHz to 15 kHz. Displacement measurements were taken at six designated locations (illustrated in Fig. 3a) using a laser Doppler vibrometer (Polytec CLV-2534).

Based on the laser displacement measurements, the deformation profile of the actuator was reconstructed at different frequencies and compared to the mode shapes generated from the ANSYS modal analysis. In Fig. 3b, mode shape sketches at 3141 Hz, 6197 Hz, and 8688 Hz from ANSYS are shown along with the measured displacement. The experimental mode shapes closely match those generated from the simulations, indicating an accurate theoretical characterization of the actuator’s physical behavior. This information is valuable for understanding the performance and operational limits of the actuator and for optimizing its design for specific applications.

### Fiber fabrication

For the waveguide, an off-the-shelf optical fiber was selected and chemically tapered to optimize its mechanical and optical properties for the scanner. As part of this process, we developed a novel method of tapering optical fibers that improves fiber flexibility, the FOV, optical beam profile, and line resolution while reducing complexity and cost.

To achieve the largest displacement and rotational angle for the fiber, the PZT optical scanner must operate at the fiber’s resonance frequencies. However, the line resolution of the scanner (when operated in a raster scan, for example) is directly influenced by the ratio of the vertical and horizontal operating frequencies. The resonant frequencies can be adjusted by optimizing the dimensions and geometry of both the optical fiber and the actuator. Specifically, matching the length of the optical fiber to the resonance frequency of the actuator maximizes the displacement of the fiber tip. Euler’s beam Eq. (1) was employed to estimate the natural frequencies of various lengths of the optical fiber. The material properties of the Corning SM600 single-mode optical fiber, which was utilized in this design, are summarized in Table S3 to provide a comprehensive reference.

1 $${f}_{n}=\frac{{\alpha }_{n}^{2}}{2\pi }\sqrt{\frac{EI}{\rho A{L}^{4}}}$$

The constants α_n_ for the first three natural frequencies are 1.875, 4.694, and 7.855. The frequency of the second resonance is approximately 6.27 times that of the first mode. However, tapering the tip of the fiber results in a lower resonant frequency than the estimated value from (1). The desired 2nd mode frequency is approximately 8 kHz to avoid the difficulties of exciting the scanner above 10 kHz as was observed in previous experiments. From the results tabulated based on Eq. 1 and summarized in Table S4, the optimized length of the SM600 fiber for the proposed scanner is calculated to be 8.4 mm.

**Fig. 4:**
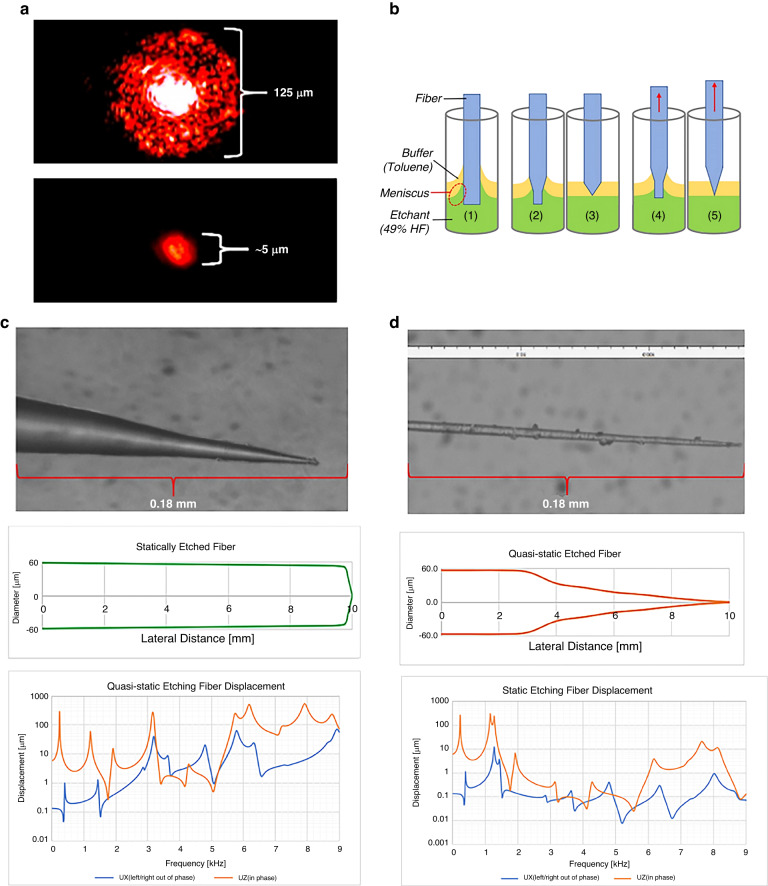
Fiber tapering process and results for static and quasistatic tip etching. **a** Comparison of fiber light transmission before (top) and after tapering. **b** Diagram of static and quasistatic etching process: (1) unetched fiber and legend, (2–3) static etching process, (4–5) quasistatic etching process depicting motion of fiber. **c** Image of the statically etched fiber tip, approximate profile, and frequency response of fiber displacement in the *X* & *Z* directions (air damping = 0.0115). **d** Image of quasistatically etched fiber, approximate profile, and frequency response of fiber displacement in the *X* & *Z* directions (air damping = 0.0115)

To remove extraneous light coupled into the cladding (Fig. 4a (1)) and refine the ‘pixel size’ of the fiber tip (Fig. 4a (2)), the fiber is tapered by chemically etching the distal end of the optical fiber to a conical profile.

In the etching process, the fiber is immersed in a solution containing 49% hydrofluoric acid (HF) and toluene, with a depth ratio of 25 mm to 10 mm. Toluene is a buffer to prevent HF vapor from overetching the fiber above the solution and reduces the HF surface tension on the fiber, as shown in Fig. 4b (1). The meniscus at the interface of the two chemicals gradually decreases as the fiber diameter shrinks, helping create the tapered profile (Fig. 4b (2), (4))^72,73^. The exact taper profile dimensions are provided in Table S5 in the Supplementary section.

An ideal optical fiber for scanning has a gradual taper to the distal end. However, the static fiber etching process tends to over-etch the fiber, and the tip breaks off when removed from the solution, as shown in Fig. 4b (3). This process leads to an extremely short conical tip of approximately 0.2 mm compared to the overall length of 8.4 mm, as illustrated in Fig. 4c.

To prevent overetching and tip breakage, a "quasistatic" process was proposed, wherein the fiber is gradually raised out of the solution by 0.5 mm every 2.5 minutes, as shown in Fig. 4b (4) and (5). This approach prevents the overetching issue, resulting in a very long and linear tapered fiber, as depicted in Fig. 4d. In this process, careful control of the immersion depths in the HF/toluene solution (initially at 25 mm/10 mm) is crucial to maintain the etchant meniscus profile.

The tapered profile of the fiber tip reduces its stiffness, leading to larger displacements when scanning. The modal analysis results, as listed in Table 2, show that the quasistatic case provides more viable operating frequencies under 10 kHz, including the highest mode under 10 kHz. This larger range of frequencies is beneficial for optimizing higher line resolution and matching the operating frequencies of the actuators to enhance the displacement of the fiber tip.

**Table 2:** Modal analysis for tapered fibers

Mode	Static (Hz)	Quasistatic (Hz)
1	1271.3	3188.7
2	1276.3	3190.3
3	7983.2	5774.5
4	8016.1	5779.4
5	10536	8907.1
6	10560	8929.1
7	22849	10500
8	22934	10531
9	29623	13170

Comparing the harmonic responses of tip displacement at various operating frequencies, the static tapered fibers show very small displacements that rarely exceed 10 μm. In contrast, the quasistatic tapered fibers exhibit much higher displacements (>100 μm) in the vertical direction (Uz) and a significant measured horizontal vibration (Ux) of 70 μm at 8.9 kHz. These results demonstrate that the quasistatic etching process is a better choice for the scanner, especially when operating at higher frequencies, as shown in Fig. 4c, d.

### Scanner experiment setup

**Fig. 5:**
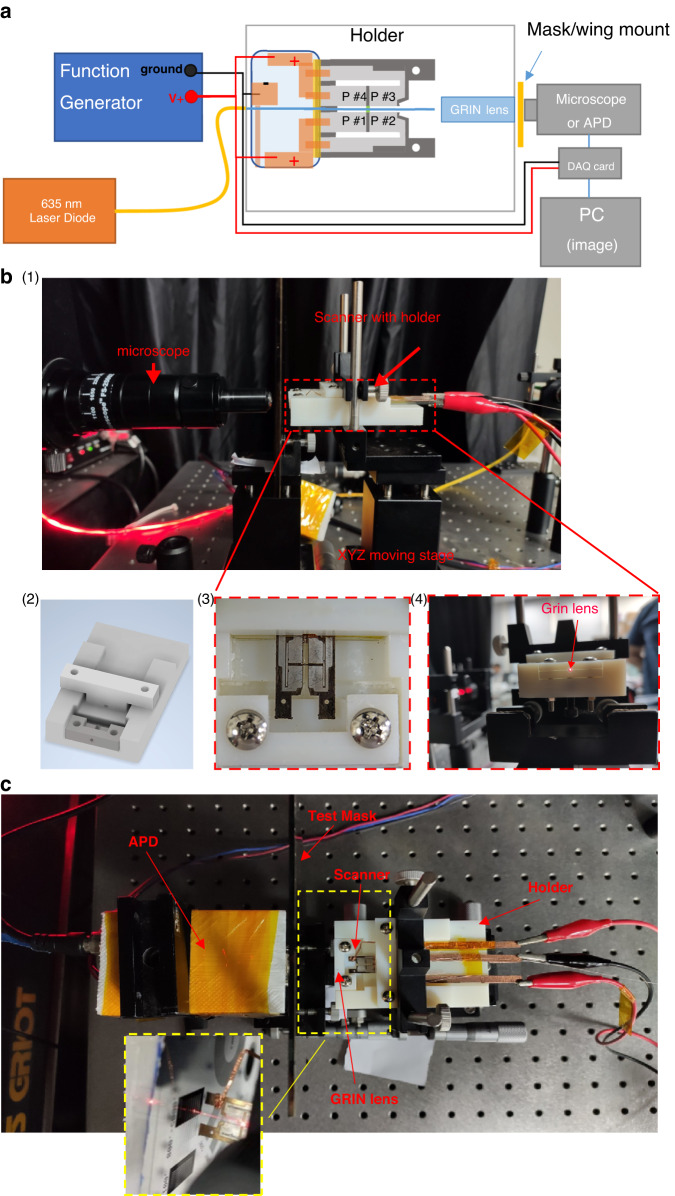
Scanner experiment setup for illumination and control tests. **a** Diagram of the image acquisition experiment setup. The scanner and GRIN lens are secured by a holder mounted to an *XYZ* moving stage to ensure alignment for both the microscope and APD image acquisition. The DAQ card is only used with APD measurements. The frequency response tests had the microscope directly observe the scanner fiber tip. **b** (1) Side view of the frequency response experiment setup showing mounting of the holder on the *XYZ* stage. (2) Holder design, (3) scanner secured in holder, (4) light output of GRIN lens secured by the holder. **c** Image acquisition test setup for the pass-through configuration. The yellow dotted window shows the top view of the device emitting light toward the test pattern on the chrome mask

The experimental setup is shown in Fig. 5, where the scanner illuminates a target with a variable zoom microscope (380x to 2500x, Ching Hsing Computer Tech, Taiwan, China) or avalanche photodiode (APD) (HAMAMATSU C12702-03) positioned to detect the emitted or transmitted light. For both the scan pattern and target illumination tests, a 50.0 × 30.0 × 1.0 mm acrylic-based photopolymer holder was designed to secure the device behind a collimating gradient index (GRIN) lens. The holder is clamped onto an XYZ micro positioner (incremental linear encoder with 2 µm resolution, Thorlabs) (Fig. 5b(1)). The holder was fabricated using a 3D printing system (Connex350, Stratasys Ltd.) (Fig. 5b(2)). The GRIN lens (4.85 mm length × 2 mm diameter GRIN lens rod. Working distance = 20 mm, beam width = 20 µm, view angle = ±30°, NA = 0.5. GRINTECH GmbH, Jena, Germany) is clamped between the upper and lower parts of the holder to focus the diverging beams from the waveguide onto a plane, as shown in Fig. 5b(3, 4). A laser diode (λ = 635 nm, 100 mW Diode Laser System, Opto Engine LLC) provides the input light to the tapered fiber via a multiaxis single mode fiber coupler system. The GRIN lens is placed 0.4 mm from the end of the fiber, resulting in an estimated beam width of 3 µm at the focal plane. The calculated angular deflection of the scanning system is approximately 5° total (2.5° on each side), which minimizes aberrations and vignette at the output. Optical tests to measure the beam profile emerging from the GRIN lens demonstrated a 3 µm beam diameter at a 2 cm focal length, matching the estimated beam width.

To observe scan patterns and actuation, a variable zoom microscope was placed near the tip of the fiber to observe the light emitted and measure displacement (shown in Fig. 5a, b(1)). The targets used in the imaging tests included line- and cross-patterned masks and a Psychodidae wing. To detect the scanned targets, an avalanche photodiode (APD) was positioned behind the test samples, as shown in Fig. 5a, c. Light from the scanner passed through the target to the APD. The sampling rate of the photodetector was set at 600 kHz, recording 75 data points per circle.

### Linear displacement test

**Fig. 6:**
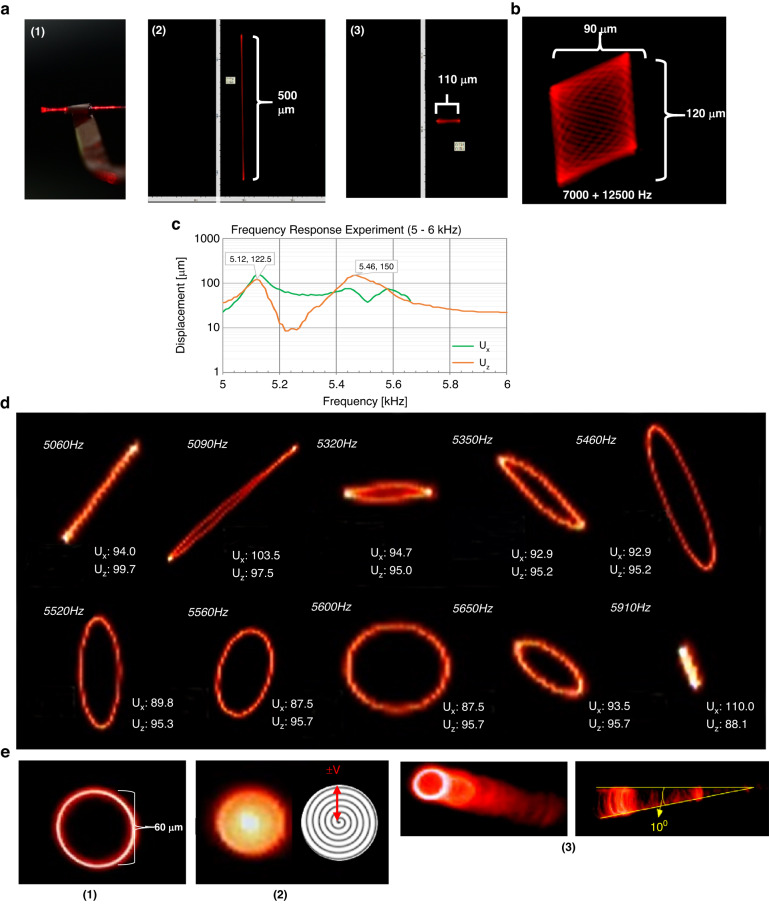
Patterns of scanner movement captured by camera. **a** 1-D scanning. (1) Side view of the vibrating tapered optical fiber with laser coupling (20VPP, 365 µm vertical displacement), (2) The largest observed vertical scan, 500 µm at 20VPP 8280 Hz, (3) the largest observed horizontal scan, 110 µm operating at 20VPP, 110° phase delay between two inputs (Fig. 5a) and 12550 Hz **b** Lissajous pattern created by driving the PZT pad pairs at different frequencies (7000 & 12500 Hz). **c** Frequency response for pad pairs driven out-of-phase. **d** 2-D scanning patterns created by nonlinear vibration from a single frequency input (units in microns). **e** Scanning method used for this paper: (1) circular scan created by operating the scanner with 6030 Hz, ±10 V in-phase triangular wave inputs, (2) spiral scan generated from circular scan with an applied 10 Hz amplitude modulation to change the radius, (3) timelapse view of the circular scan captured in motion, and a side view of its corresponding fiber displacement angle (10°)

The scanner has several potential modes of operation, as demonstrated in Fig. 6. For linear actuation, Fig. 6a(2, 3) shows the displacement in the vertical and horizontal directions. Figure 6b shows a Lissajous scan generated by driving the two sets of PZT pads (Fig. 5a, pads 1 & 2 connect to one input channel, pads 3 & 4 connect to the second channel) at different amplitudes, frequencies, and phases.

First, 1D scanning operations were performed using a 14.6VPP triangular wave input to generate the frequency response of the scanner’s vertical displacement (Uz). The comparison of the experimental results and simulation results for the vertical displacement is shown in Fig. 3d. When comparing the four indicated frequencies, the deviation ranges from 4.5% to 12%, which we considered acceptable considering the lack of refinement in the fabrication and assembly process. The lower frequency shifts and deviations are likely due to manually epoxying the fiber to the actuator, as excess epoxy could change the free-end length of the fiber. The largest simulated vertical displacement occurs at approximately 7917 Hz, which was confirmed experimentally with the maximum 500 μm vertical displacement observed at 20 VPP and 8280 Hz (Fig. 3a (2)). The largest horizontal displacement, 110 µm, was observed operating at 20VPP with a 110° phase delay between the two inputs (Fig. 5a) at 12550 Hz (Fig. 6a (3)).

With inputs of 7000 Hz and 12500 Hz, the optical scanner can produce a Lissajous scanning area of approximately 90 × 120 μm^2^ (Fig. 6b). While the Lissajous provides an ample FOV without sacrificing line resolution, endoscopic probes usually have a cylindrical enclosure, making circular or spiral scan patterns preferable to fully utilize the available aperture. Furthermore, the Lissajous pattern requires tuning two independent frequencies, amplitudes, and phases to compensate for nonlinear effects caused by imperfect manufacture of the scanner components.

### Nonlinear displacement

A novel method for generating circular scan patterns was achieved by harnessing the naturally occurring nonlinear vibrations within the scanner. Ideally, the scanner fiber would have a perfectly symmetrical cross-section with uniform natural frequencies in both directions, resulting in highly linear vibration responses to excitation. However, due to imperfections in the fiber etching process and system assembly, slight deviations can occur in the fiber geometries and natural frequencies, as depicted in Fig. 6c. These scan patterns are attributed to the nonlinear vibrations caused by the physical construction of the scanner, most notably an asymmetrical cross-section for the optical fiber and epoxy on the center arm. These imperfections lead to a bifurcation in the frequency response when a large one-dimensional vibration (two in-phase inputs at a single frequency) is introduced to the system. Consequently, various two-dimensional scan patterns, such as lines, ovoids, and circles, are produced (as illustrated in Fig. 6c, d, e) based on the difference in magnitude of the orthogonally generated vibrations. This characteristic enables the two-dimensional scan patterns to change (as shown in Fig. 6e) by varying a single operating frequency (Fig. 5a).

**Fig. 7:**
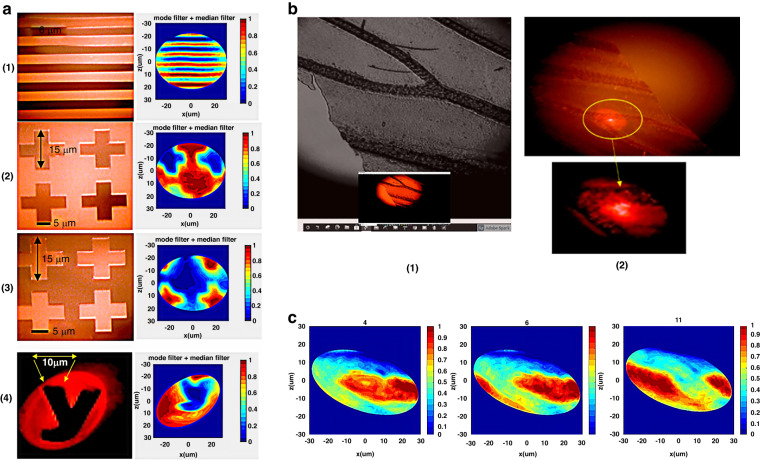
Reconstructed image results. **a** Masks (photographs left) and images reconstructed by the APD (right), (1) line pattern with line and gap widths of 5 µm, (2) solid cross pattern with 15 µm length and 5 µm gaps, (3) hollow cross pattern with 15 µm length and 5 µm gap, (4) letter “y” with 10 μm line width. **b** Psychodidae wing illumination. (1) Microscopy image of the wing, (2) area of the wing illuminated by the scanner. **c** Images captured by the APD, showing wing structures as the FOV moves from right to left

From simulations and experiments, we found that our system excites nonlinear vibrations in the 5–6 kHz (Fig. 6e) and 7–9 kHz regions (Fig. 6a). The shape of the circular scan and the amplitude of the nonlinear vibration can be determined from the frequency response in the two orthogonal directions, as shown in Fig. 6c. By exploiting this nonlinear behavior, only a single input is necessary for 2D scanning, as shown in Fig. 6.

In operation, the device operates from a single +10 V 8005 Hz triangular wave input with a 10 Hz AM frequency, signifying that the scanner tip will traverse 400 circles in 0.05 seconds. In this case, the scanning pattern appears slightly elliptical (Fig. 6a, b) due to the single input nonlinear vibration.

During testing, the largest displacements occurred at approximately 6 kHz, with the maximum circular scan area created at 6360 Hz with a diameter of approximately 60 μm, as shown in Fig. 6e(1). By adding amplitude modulation to the circular scan input signal, a spiral scan pattern can be created, as shown in Fig. 6e(2, 3), with a modulation frequency of 10 Hz.

### Image acquistion

Several targets were illuminated using a spiral scan method (Fig. 6e(2)), with an APD used to detect the transmitted light for image reconstruction, as shown in Fig. 7a. The target patterns include a line pattern with 5 μm line and gap widths (1), positive (2) and negative (3) cross patterns with 15 μm lengths and 5 μm gaps, and a “y” (4) with a line thickness of 10 μm. Additionally, a Psychodidae wing pasted on a transparency mask was tested to study biological structures (Fig. 4b, c). The nonlinear vibration-generated spiral scan was chosen for use in experiments because it provided the simplest actuation, wherein the scanner demonstrates a line width resolution down to 5 μm.

### Image reconstruction

Designing a MEMS scanner for micro imaging presents technical challenges, such as generating a distortion-free scan pattern with sufficient speed to overcome motion artifacts caused by live physiological movements. The data collected during a scan are a 3 by n matrix containing the time, driving voltage magnitude, and measured light intensity. A four-part MATLAB program reconstructs the image through data chain segmentation, signal normalization, data mapping, and postprocessing.

**Fig. 8:**
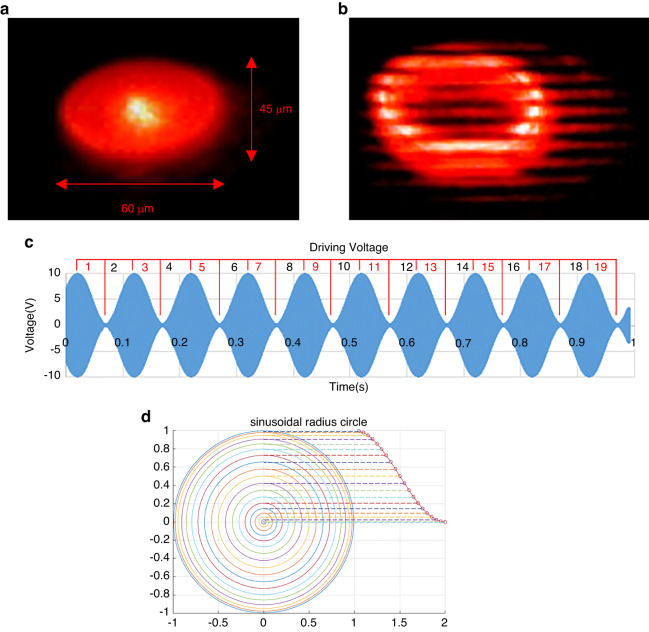
Spiral scan and frame isolation for image reconstruction. **a** Dimensions of the elliptical shaped spiral scan pattern, operated at 8005 Hz with 10 Hz AM for parallel lines with 5 μm linewidth and 5 μm gap act as target pattern (see Fig. 7a). **b** Microscopy image of the elliptical scan over parallel lines. The AM is temporarily turned off for a clear picture. **c** Isolating data frames from the driving voltage. A frame is defined from the maximum voltage to the minimum voltage and from minimum to maximum so that the frame rate is twice the AM frequency. **d** Schematic of the sinusoidal radius concentric circle. The radii of the concentric circles change sinusoidally instead of linearly

The data chain segmentation creates frames by finding the local maxima and minima of the driving voltage to define the start and end of a frame. The driving voltage corresponds to the radial displacement of the tip from the center (0 V) position; thus, within one AM period (0.1 s), two complete frames occur as the tip is spun in a circle by the 8005 Hz primary signal (Fig. 8a, b).

After the data are segmented, the phase of the target image intensity and the reference intensity (reference intensity is a preexisting dataset from calibration) are aligned, allowing for normalization. After normalization, the absolute intensity was converted to relative intensity (Fig. 8c).

The relative intensity values of each frame are segmented into 74 or 75 points corresponding to the 8005 Hz (0.125 ms) period since only the first half of the cycle was used for imaging. Each point represents one displacement radius in the spiral scan. For data mapping, a parametric equation of an ellipse is applied to every data segment to plot concentric circles or ellipses, with the spacing of the circles changing sinusoidally and following the trend of the amplitude modulation (Fig. 8d).

The scanned image is plotted to a 601 x 601 pixel image scaled to a 60 × 60 μm^2^ area, with the intensity represented by either the color or height of each data point. Interpolation is used to fill the space between data points. However, the resulting image may contain noise and pincushion distortion.

To address measurement noise, mode and median filters are applied to the image. The mode filter replaces pixels with the most frequently occurring pixel value within a certain window size (3 x 3 pixels in this case), while the median filter replaces pixels with the middle pixel value selected from a certain window size (15 x 15 pixels in this case). Both filters are effective for noise removal while preserving edge information.

Several sources of distortion are also corrected in the program. First, the pincushion distortion introduced by the GRIN lens is manually corrected through trial-and-error, as observed from the scanned image for parallel lines with the same gap and linewidth. Second, a software delay is introduced to account for the mechanical delay between the driving voltage and the actual vibration of the fiber tip, ensuring accurate positioning of the scan location to the driving voltage. After these corrections, the scan data are properly divided for further processing.

Ultimately, we successfully reconstructed the results of real images from the scanning targets using the described process. We tested various patterns, including line patterns, cross patterns, and a letter “y”, on the chromium mask, as well as a Psychodidae wing pasted on the transparency mask. Figure 7a displays the real images and reconstructed results of these chromium mask scanning targets, while Fig. 7b shows the biological structure of the wing. Based on initial testing, we propose that this system can achieve a line scan with a pattern of 5 µm line and gap width, as well as a cross-pattern scan with 15 µm length and 5 µm gap. We also demonstrated that a biological sample can be imaged using the current system.

## Discussion

To investigate the novelty of our approach for designing, manufacturing, and controlling a MEMS scanner, we reviewed competing technologies and approaches. We conducted a detailed review of over 20 papers and highlight the results of seven that exemplify the current state of the art. Table 3 lists a comprehensive comparison of scanning-based fiber technology, MEMS, and our micro imaging technologies^74–80^.

**Table 3:** Summary of various MEMS-based scanning micro imaging systems

Scanning Methods	Actuator/System Size (mm^3^)	Scanning Frequency (Hz)	Field of view (^o^ or μm^2^)	Spatial/line resolution (μm/)	Operating Condition (V)	Reference
Electrostatic MEMS Micro mirror	3.0x3.0 (actuator)/10x5(OD) (system)	>900	±6°	<5 µm(optical)/12μm transverse resolution/150x120pixels	>+100 V (for both V and H input, linear vibration)	Aguirre et al.^74^
Electromagnetic MEMS Micro mirror	2.4x2.9(actuator)/12x2.8(OD) (system)	>350	±30°	5 µm(optical) 23 µm transverse resolution/60x40 pixels	±1.2 V (V) and ±4 V(H) input, linear vibration	Kim et al.^75^
Piezoelectric MEMS Micro mirror	7x5(actuator)^a^/N.A. (system)	1000 (0.6x0.8 mirror size)	±7°	N.A.	+20 V (for both V and H input, linear vibration)	Gilchrist et al.^76^
Electrothermal MEMS Micro mirror	1.5x1.5(actuator)/25x4 (OD) (system)	46	±17°	20 µm/25x25pixels	+1.7 V (for both V and H input, linear vibration)	Xu et al.^77^
Scanning fiber endoscope	4x0.45(OD) (actuator)/ 9x1.2(OD) (system)	11000 (V&H with phase shift to generate circular scan)	75°	2 µm/>500lines	<+20 (for both V and H input, linear vibration)	Seibel et al.^78^
Electrothermal MEMS fiber scanner	1.28x7x0.44 (device)/ 1.28x20x0.44 (system)	218.4(V)/239.4(H) Lissajous scan	451 × 558	>20 µm (based on the scanning image)	+16 (for both V and H input, linear vibration)	Seo et al.^79^
Fully Integrated MEMS waveguide Scanner	3.0x3.0x0.5(actuator)/8.0 × 8.0 × 1.0 (system)	201(V)/20(H) Raster scan	4.1° × 1.1°/130 × 19	20 μm	+150 V input (for bth V and H input, non resonating linear vibration)	Wang et al.^35^
PZT-Actuated Fiber MEMS Scanner	7.0x6.0x0.07(actuator)/ 17x10x0.07 (system)	8005 (Circular scan)	10°/60 × 60	3 µm at fiber tip 5 µm after GRIN lens/800lines	+10 V (Single input, nonlinear vibration)	Wang et al.

^a^Only utilizes a single mirror, and would require two mirrors to create orthogonal direction scanning

Among MEMS-based micro imaging systems, micro mirror-based actuated systems currently dominate the field. Four prevalent driving mechanisms are commonly employed: electrostatic, electromagnetic, piezoelectric, and electrothermal actuation. In the present comparison, we primarily focus on techniques deployed in a series of endoscopic probes^74–77^.

Aguirre et al. presented an endoscopic OCT probe based on an electrostatic MEMS mirror^74^. Their study showcases a gimbaled 2D MEMS mirror design that utilizes angular vertical comb actuators, allowing for larger scan angles compared to other vertical comb drives of similar dimensions. The mirror has a circular aperture with a 1 mm diameter, and the device itself has a footprint of 3 × 3 mm². With voltages exceeding 100 V, the mirror achieves mechanical scan angles of ±6° on both axes. The side-view probe, housed in an aluminum holder, has an approximate diameter of 5 mm^74^. Integrated into a spectral domain OCT system, the probe achieves a scanning rate of 4 frames/sec over a range of 1.8 × 1.0 × 1.3 mm. To demonstrate its capabilities, the authors successfully obtained 3D images of a hamster cheek pouch using the probe.

In a study by Kim et al., a 2D electromagnetic MEMS mirror-based endoscopic OCT probe was demonstrated^75^. Their design incorporates a 2D gimbaled mirror with a magnet attached to the mirror plate and wire-wound coils inside the probe body for each scan direction. The mirror plate has dimensions of 0.6 × 0.8 mm, and the device footprint measures 2.4 × 2.9 mm. The inner and outer axes achieve ±30° optical scan angles with driving voltages of ±1.2 V and ±4 V, respectively, corresponding to currents of 50 mA and 100 mA. With an SD-OCT system, a probe with a diameter of 2.8 mm and length of 12 mm was demonstrated. Using ±2.8 V and ±0.8 V voltages on the inner and outer axes, they obtained 3D images of fingertips at a rate of 18.5 frames/s, covering a lateral scan range of 1.5 × 1 mm. The total power consumption was 150 mW. However, a drawback of electromagnetic mirrors in endoscopic applications is their high power consumption and the need for external magnets, which complicates the packaging process and limits further miniaturization. Additionally, electromagnetic interference is a concern to be addressed.

Piezoelectric actuation is another approach that is used in the design of MEMS-based micro mirror imaging, which leverages the piezoelectric effect to induce bending motion by applying an electric field across a piezoelectric material such as lead zirconate titanate (PZT). Piezoelectric actuators typically consist of a sandwich structure of metal/PZT/metal or double-layered PZT materials^41–52^. In a reported study^76^, a piezoelectric MEMS-based OCT probe was developed. The researchers fabricated piezoelectric MEMS mirrors with aperture sizes of 600 × 840 μm and 840 × 1600 μm. These mirrors achieved mechanical scan angles of up to ±7° and exhibited a resonant frequency of up to 1 kHz (as shown in Table 3).

An electrothermal actuation-based MEMS OCT probe was presented by Xu et al.^77^ Researchers have developed MEMS mirrors equipped with straight and curled-shaped electrothermal bimorph actuators composed of aluminum and silicon. The largest mechanical deflection achieved was 17° at an operating voltage of approximately 1.3 V^77^ (as indicated in Table 3). The device featured a 500 μm diameter mirror aperture situated on top of a 1.5 × 1.5 mm chip. Notably, the mirror exhibited a linear relationship between the driving voltage and scan angle beyond the initial critical voltage, and its 3 dB cutoff frequency was measured at 46 Hz. In the probe assembly, the silicon optical bench (SiOB) methodology was employed to achieve self-alignment of the optical components, while the electrical connections from the MEMS mirror to the copper wires on the substrate were established using solder balls. With a diameter of less than 4 mm and a rigid length of approximately 25 mm^77^, the probe demonstrated its potential for endoscopic OCT imaging.

Among these actuation mechanisms, electrostatic actuation is fast and energy-efficient, but its necessary high driving voltages limit its suitability for imaging internal organs. Electromagnetic actuation enables large scan angles at low voltages, but permanent magnets complicate miniaturization. Piezoelectric MEMS mirrors offer fast response, low power consumption, and wide bandwidth but need to address issues of tilting, hysteresis, and charge leakage. Electrothermal actuation achieves large scan angles at low voltages with a high fill factor but has a slower thermal response than other techniques. Overall, electrothermal MEMS mirrors are preferred for micro imaging scanners. For a more detailed analysis of this topic, refer to reference^80^.

The piezo-electrical tube-based scanning fiber endoscope (SFE)^78^ and electrothermal MEMS fiber scanner^79^ technologies have found application in micro imaging, particularly in the field of molecularly targeted imaging. In the SFE, a multiplexed excitation laser beam is directed through a single-mode fiber integrated into custom-made thin-wall piezo tubing with a small collar. The SFE probe incorporates a micro-optic lens group with a small outer diameter. To collect fluorescent emission light, multimode fibers with a numerical aperture (N.A.) of 0.63 (outer diameter 250 µm) surround the tubing jacket. After passing through a condensing lens, the light is detected using photomultiplier tubes. The detection system includes longpass filters and notch filters to minimize the impact of reflective light from the collection multimode fibers. The fiber scanner utilizes a spiral scanning pattern with a divergence angle of approximately 70°^78^. While our current design shares similarities with the scanning fiber concept, their actuator employs an off-the-shelf 4 mm long and 450 µm (OD) tubular piezo tube, which may introduce complexities in the assembly process.

The electrothermal fiber MEMS scanner^79^ incorporates a distinctive feature that achieves separation of resonant scanning frequencies between the lateral and vertical directions of a single optical fiber. This separation enables the realization of Lissajous scanning during the resonant motion. The microactuator has a compact footprint dimension of 1.28 × 7 × 0.44 mm^3^. Specifically, the resonant scanning frequencies for a 20 mm long optical fiber are 239.4 Hz and 218.4 Hz in the lateral and vertical directions, respectively. With a 16 Vpp pulse train, the full scanned area covers 451 × 558 μm^79^.

Our previous research introduced a fully integrated MEMS-based nonresonating 2D mechanical scanning system using a 1-D push-pull actuator^35^. This system achieved broadband single-mode operation (λ = 0.4 μm to 0.65 μm) with a vertical frequency of 201 Hz and a horizontal frequency of 20 Hz. It offered an FOV from 0.019 to 0.072 radians in both directions, which was unaffected by fabrication uncertainties. Two fundamental resonances were observed at 201 Hz and 536 Hz in the vertical and horizontal directions, respectively, with corresponding displacements of 130 μm and 19 μm (or FOVs of 0.072 and 0.0105 radians) at +150 V input. A GRIN lens focused the output beam to a 20 μm diameter at the focal plane. However, the design’s 7-layer double wafer structure made its fabrication extremely complex.

The current proposed fiber scanner offers significant performance improvements compared to existing MEMS mirrors and fiber scanners. The scanner utilizes high-speed piezoelectric actuators with a unique push-pull bimorph actuator design, enabling precise and rapid motion control for faster scanning. Although our current device is slightly larger and has a limited FOV, we can further enhance its performance by making minimal modifications to the fiber geometry and dimension to achieve larger deflection angles without increasing actuation requirements. One of the main advantages of our design is its exceptionally high operating frequency of 8004 Hz, which is 2 to 3 times higher than those of the best-performing imaging systems in Table 3. Furthermore, our spatial resolution (5 μm) is comparable to that of existing fiber-based micro imaging systems. The bimorph piezo actuator design is a novel approach that improves mechanical actuation with relatively low voltage operation, and further enhancements can be achieved through geometric design modifications (which are currently underway). Our system offers greater simplicity of operation than do other MEMS or fiber scanners, as it requires only single-direction actuation to generate the desired 2D spiral scanning motion due to its nonlinear vibration excitation.

## Conclusion

In this paper, we have successfully demonstrated image illumination and acquisition by a novel MEMS scanner system comprising aerosol-jetted PZT bimorph push-pull MEMS actuators driving a tapered optical fiber waveguide. Details of the design, fabrication, and testing have been presented. Preliminary tests prove that the push-pull actuator concept works as intended and is capable of creating 2D scanning motion using 1D actuators.

One of the greatest advantages of the proposed scanner design is that larger vibrations at higher scanning frequencies can be achieved through simple modifications to the scanner’s mechanical design. We accomplish these modifications by tapering the tip of the optical scanner fiber and selecting matching fiber and actuator natural frequencies. Our PZT-based optical scanner achieves a scanning frequency of up to 8 kHz. The scanner’s mechanical and optical parameters were carefully analyzed and optimized to determine the best line resolution and FOV. These optimization parameters were experimentally verified and realized 3 µm line resolution and a 10° FOV. In resolution tests, the PZT optical scanner resolved small line images down to 5 µm. For 2D images, the resolution is currently limited to 15 µm in solid features and 5 µm in transparent gaps. The PZT optical scanner shows great promise in scanning applications, with the potential to become a microscopic imaging device with further development. This device represents a critical step toward the development of a full illumination/acquisition MEM scanner with applications in endoscopy and microscopic imaging.

### Supplementary information

Supplemental Material
