# Supplementary material for: Mirrorless MEMS imaging: a nonlinear vibrational approach utilizing aerosol-jetted PZT-actuated fiber MEMS scanner for microscale illumination
Source: Microsyst Nanoeng. 2024 Jan 22;10:13. doi: 10.1038/s41378-023-00646-5 (PMC10800347; doi:10.1038/s41378-023-00646-5)
Supplement: Supplementary file 1 — Supplemental Material [file 41378_2023_646_MOESM1_ESM.docx]

**Supplementary Information**

**Mirrorless MEMS Imaging: A Nonlinear Vibrational Approach Utilizing Aerosol-Jetted PZT-Actuated Fiber MEMS Scanner for Micro-scale Illumination**

Wei-Chih Wang^1,2,3,4*^ Ming-Yao Li^1^, Kuan-Chang Peng^1^, Yi-Feng Hsu^1^, Benjamin Estroff^3,4^, Pao-Yun Yen^1^, David Schipf^3^, Wen-Jong Wu^5^,

^1^ Department of Power Mechanical Engineering, National Tsing Hua University, No. 101, Section 2, Kuang-Fu Road, Hsinchu, Taiwan 30013, China

^2^ Institute of NanoEngineering and Microsystems, National Tsing Hua University, No. 101, Section 2, Kuang-Fu Road, Hsinchu, Taiwan 30013, China

^3^ Department of Mechanical Engineering, University of Washington, Seattle, WA. 98195, USA

^4^ Department of Electrical Engineering, University of Washington, Seattle, WA. 98195, USA

^5^ Department of Engineering Science and Ocean Engineering, National Taiwan University, Taiwan, China

*abong@uw.edu

**Supplementary Tables**

| **Table S1** Material properties of scanner components | | | |
| --- | --- | --- | --- |
| Material | Density  (kg/m^3^) | Young’s Modulus (kg/ms^2^) | Poisson’s ratio |
| Stainless-steel | 7930 | 1.93E+11 | 0.25 |
| PZT | 7500 | N/A | N/A |
| Optical fiber | 2410 | 7.06E+10 | 0.17 |
| Epoxy | 1250 | 3.5E+6 | 0.33 |

| **Table S2** Piezoelectric and Elastic Compliance Matrix | | | | |
| --- | --- | --- | --- | --- |
| Piezoelectric Coefficient | Value | Elastic  Coefficient | Value |  |
| d13 | -5.3512E-12 | SE11 | 1.64E-11 |  |
| d23 | -5.3512E-12 | SE12 | -5.74E-12 |  |
| d33 | -15.7853E-12 | SE13 | -7.22E-12 |  |
| d42 | 12.2947E-12 | SE33 | 1.88E-11 |  |
| d51 | 12.2947E-12 | SE44 | 4.43E-11 |  |
|  |  | SE66 | 4.75E-11 |  |

| **Table S3** SM600 Optical Fiber Properties | | |
| --- | --- | --- |
| Variable | Value | Unit |
| *E*, Modules of elasticity | 7.06E+10 | N/m^2^ |
| *I*, Moment of inertia | 1.2E-17 | m^4^ |
| *ρ*, Density | 2.41E+3 | Kg/m^3^ |
| *A*, Cross sectional area | 1.23E-8 | m^2^ |
| *L*, Length | 0.0084 | m |

| **Table S4** Effective Length of SM600 vs Resonant Frequency | | |
| --- | --- | --- |
| Length of fiber (mm) | 1^st^ mode (Hz) | 2^nd^ mode (Hz) |
| 8 | 1478.2 | 9264.4 |
| 8.5 | 1309.4 | 8206.5 |
| 9 | 1168.0 | 7320.0 |

| **Table S5** Profile of a chemically-etched fiber using the quasi-static etching. | | |
| --- | --- | --- |
| **Length Position**  **[mm]** | **Diameter**  **[μm]** | 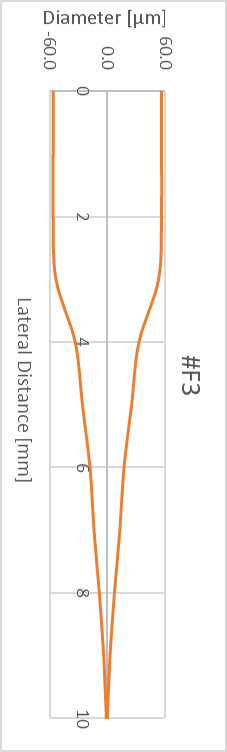 |
| **0** | 113 |  |
| **1** | 113 |  |
| **2** | 113 |  |
| **3** | 107 |  |
| **4** | 66 |  |
| **5** | 51 |  |
| **6** | 36 |  |
| **7** | 27 |  |
| **8** | 16 |  |
| **9** | 7 |  |
| **10 (tip)** | 1 |  |

**Supplementary Figures**

The primary objective of the annealing process is to enhance the alignment and orientation of the crystal domains within the PZT material, improving the piezoelectric properties of the device. Additionally, annealing serves to mitigate dielectric losses by optimizing the material's dielectric constant and minimizing imperfections within the crystal structure, resulting in a smoother surface. Moreover, this procedure guarantees that the PZT material attains increased stability and long-term reliability, rendering it suitable for extended applications.

When annealing, sudden changes in temperature can cause thermal shock and cracking of the PZT, so the temperature is gradually ramped up from room temperature to 520^o^C over four hours. This temperature is held for three hours, then cooled to room temperature over the next 25 hours.


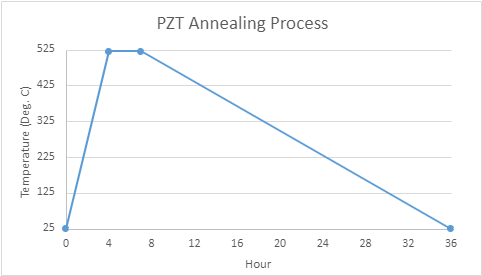


| Time (hr) | Temperature (C°) |
| --- | --- |
| 0 | 25 |
| 4 | 520 |
| 7 | 520 |
| 36 | 25 |

**Fig. S1.** Annealing process temperature profile.

**Supplementary Calculations**

Calculation of dielectric constant

After forming the four bimorph actuators, the capacitance of each PZT film pad pair was measured before and after poling. From this and the pad dimensions, the relative dielectric constant can be calculated from:

$C=\frac{\varepsilon*W*L}{d}$ (S1)

Which rearranges to:

$\varepsilon=\frac{C*d}{W*L}$ (S2)

Where W=2596 µm, L=3575 µm, d=6.2 µm (based on the thickness measured by profilometer).

Capacitance values before and after poling are recorded in the following table:

**Table S6**  Measured capacitance values for electrodes

| Electrode # | C_1_ Capacitance before poling (nf) | C_2_ Capacitance after poling (nf) |
| --- | --- | --- |
| 1 | 19.8 | 18.1 |
| 2 | 16.3 | 14.9 |
| 3 | 16.9 | 15.3 |
| 4 | 20.4 | 19.3 |
| Average | 18.4 | 16.9 |

From this, the dielectric constant of C_1Average_ =1.226E-08 F/M and C_2Average_=1.1290E-08 F/M.

To find the relative dielectric constant ε_r_, divide by the vacuum permittivity ε_0_=8.85E-12 F/M:

$\varepsilon_{r}=\frac{\varepsilon}{\varepsilon_{0}}$ (S3)

Before poling, the ε_r_=1385.2. After poling, ε_r_=1275.7.
